# Kinetics study of anodic electrophoretic deposition for polytetrafluoroethylene (PTFE) coatings on AZ31 magnesium alloy

**DOI:** 10.1186/s13065-022-00884-0

**Published:** 2022-11-12

**Authors:** Qing Xiang, Jiyao Qin, Taihong Qin, Lu Chen, Daixiong Zhang

**Affiliations:** 1 School of materials and architectural engineering, Guizhou Normal University, Guiyang, 550001 People’s Republic of China; 2 Chongqing Western Water Resources Development Co. LTD, Chongqing, 400039 People’s Republic of China; 3 Administrative Committee of Chongqing Tongnan HighTech Industrial Development Zone, Chongqing, 402660 People’s Republic of China; 4 College of Environment and Resources, Chongqing Key Laboratory of Catalysis and New Environmental Materials, Chongqing Technology and Business University, Chongqing, 400067 People’s Republic of China

**Keywords:** Anodic electrophoretic deposition, PTFE, Coatings, Kinetics

## Abstract

Electrophoretic deposition (EPD) coating has become a hot topic due to its simple experiment, wide application, and wide material range. In this study, the PTFE coating was successfully prepared by electrophoretic deposition through the systematic study of electrophoretic deposition kinetics. In particular, in the dispersion system with ethanol as solvent, Nafion and NaOH were simultaneously added as additives to obtain a beneficial synergistic effect on PTFE electrophoretic deposition. And the best additive scheme is: when the concentration of PTFE was 6 g·L^− 1^ and the deposition time was increased to 20 min, adding 0.10 g·L^− 1^ Nafion and 0.10 mM NaOH simultaneously. Compared with the scheme with Nafion being only additive, the addition of NaOH can improve the deposition rate from 0.16 mg·cm^− 2^ to 0.98 mg·cm^− 2^, and the deposition rate increases by about 6 times. According to electrophoretic deposition kinetics, there is an obvious critical transition time between linear and parabolic regions in the preparation of the coating. Prolonging the arrival of critical transition time is beneficial to effectively achieve stable growth of the coating in a longer time. It is found that a more ideal additive can not only increase the deposition rate of coating, but also significantly accelerate the arrival of critical transition time. Meanwhile, the deposition voltage also has an important influence on the critical transition time. Increasing the voltage can improve the deposition speed but shorten the critical transition time. Therefore, the application of deposition voltage needs to strike a balance between deposition rate and critical time point. The optimal deposition conditions proposed in this work are: deposition voltage 60 V, deposition time 20 min, additive 0.10 g·L^− 1^ Nafion and 0.10 mM NaOH.

## Introduction

Polytetrafluoroethylene (PTFE) with coating formation has attracted considerable attention due to its peculiar characteristics such as excellent corrosion resistance, high and low temperature resistance, aging resistance, low friction, dielectric property, non-viscosity, physiological inertia, and has been widely used in the field of chemistry, machinery, electronic, construction, medical, etc. [1–4]. Therefore, it is desirable to develop the synthetic method for PTFE coatings. Same as other preparation methods of fluoropolymer coating [5–7], many workers have also made a lot of research in exploring the preparation of PTFE coating. For example, Yu et al. [8] have deposited PTFE coatings onto aluminum plates via electrostatic attraction self-assembly method. Bansal et al. [9] have produced PTFE coatings with hydrophobic performances by sintering process on a hydro-machinery steel SS410. Zhang et al. [10] have reported that the PTFE coatings deposited on a glass substrate exhibits superhydrophobicity using supercritical carbon dioxide (sc-CO_2_) by keeping PTFE coatings on and the glass substrate being coated under sc-CO_2_ at a certain temperature and pressure for a sustained period.

Currently, other technologies used to prepare coating on Mg alloys include chemical conversion coating [11–13], etching [14], sol–gel, [15], electrodeposition [16], etc. Moreover, electrophoretic deposition (EPD) technology has been widely concerned by researchers [17–20] due to its advantages of simple equipment, convenient method, low cost, uniform films, adaptive substrates with complex and diverse shapes, and strong competitiveness and development prospect in coating. However, few works have discussed the preparation for PTFE coatings by EPD method.

In this work, a PTFE coating was fabricated on the surface of AZ31 magnesium alloy sheets by EPD. And the microstructure and morphology of PTFE coating were analyzed. In order to keep the coating composition being in a stable control zone with prolonging deposition time, the experimental data of EPD deposition kinetics were given.

## Materials and methods

### Reagents and materials

Polytetrafluoroethylene (PTFE, < 1 μm, 99.9%) was purchased in Aladdin Industrial Co., (China). Nafion (5 wt% dissolved isopropanol) was purchased from Alfa Aesar Chemical Co., (China). Ethanol and sodium hydroxide (NaOH) were purchased from Sinopharm Chemical Reagent Co. LTD. All reagents were of analytical grade and used without further purification. AZ31 magnesium alloy sheets (99.95%) were used as electrode materials. Double distilled water was used in the experiments.

### Fabrication of PTFE coatings

AZ31 magnesium alloy sheets were 50 mm × 30 mm × 1 mm then wet ground with silicon paper from 400 # to 1000 # grid for anode and cathode electrodes materials. During EPD, the two electrodes were immersed vertically in 100 mL treated suspension with the distance between electrodes being fixed at 1.0 cm.

In all experiments, the suspensions were made by mixing PTFE particles into 100 mL ethanol solvent which contains certain amount of Nafion and NaOH. The suspension was then sonicated for 20 min using an ultrasound (Kq5200DE Kunshan Ultrasonic Instrument Co., Ltd. China) with 200 W, which was conducive to restraining the fragmentation and the agglomeration of particles in suspension.

The EPD was performed under strengths of 30 ~ 120 V·cm^− 1^ when applied in different fields, and the deposition time varied from 0 to 30 min with different additive (i.e. Nafion or NaOH) concentrations. Then the anode with PTFE coatings was removed from the suspensions and dried for 30 min at 373 K.

In this work, the deposition quantity per unit area is used for the deposition mass, that is, by accurately measuring the area of the deposition coatings, the deposition mass is divided by the deposition area to obtain the deposition mass per unit area (unit: mg·cm^− 2^). And an electronic balance was used to accurately weigh to 0.10 mg.

### Characterization

The zeta potential of PFTE was measured by zeta-sizer (Nano ZS90, Malvern Inst.) The microstructure of coatings was examined by Fourier transform infrared spectroscopy (FI-IR) with a diffractometer (Tensor 27, Bruker, Germany). The surface morphologies of the coatings were examined by scanning electron microscopy (SEM) (Gemin 300, Zeiss, Germany) with an accelerating voltage of 20 kV.

## Results and discussion

### Effect of Nafion on suspension stabilization

**Fig. 1:**
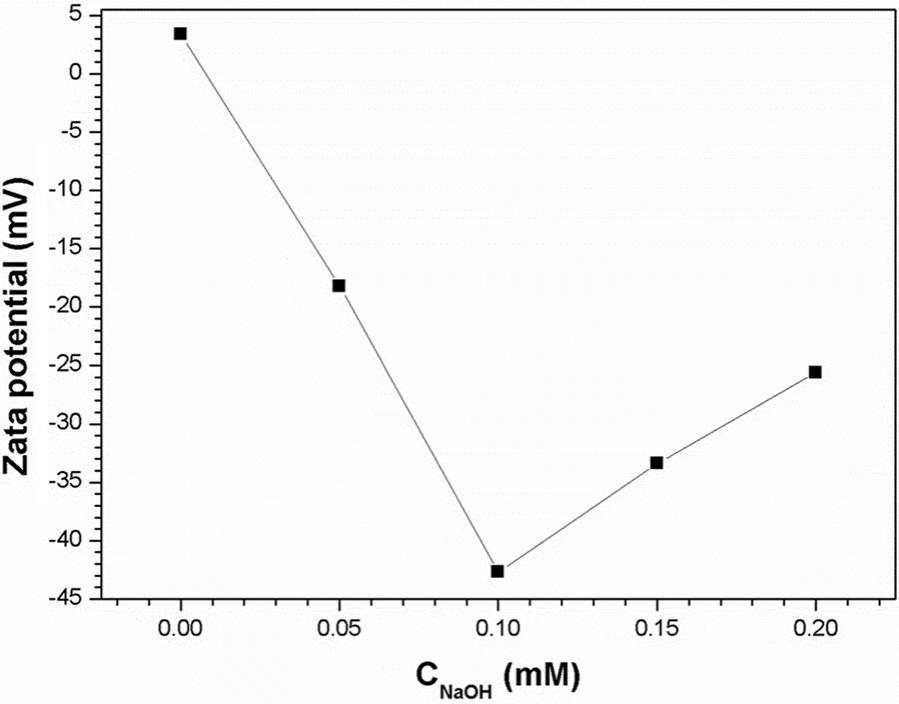
The concentration of NaOH as the functions of the zeta potential

The stability of the ethanol suspension depends on the Zeta potential of its solution. Figure 1 shows the zeta potentials of PFTE particles are displayed as the function of the amount of NaOH the with a simultaneous addition of 0.10 g·L^− 1^ Nafion. The concentration of PTFE is fixed at 6 g·L^− 1^. With the addition of NaOH, the zeta potential of the suspension becomes negative. The absolute value of zeta potential of the suspension increases with the increase of NaOH concentration. However, when the concentration of NaOH exceeded 0.10 mM, the zeta potential tend to be stable and present a downward trend. The reason may be that the surface modification process of PTFE particles has become saturated.

### Electrophoretic assembly of PTFE particles

**Fig. 2:**
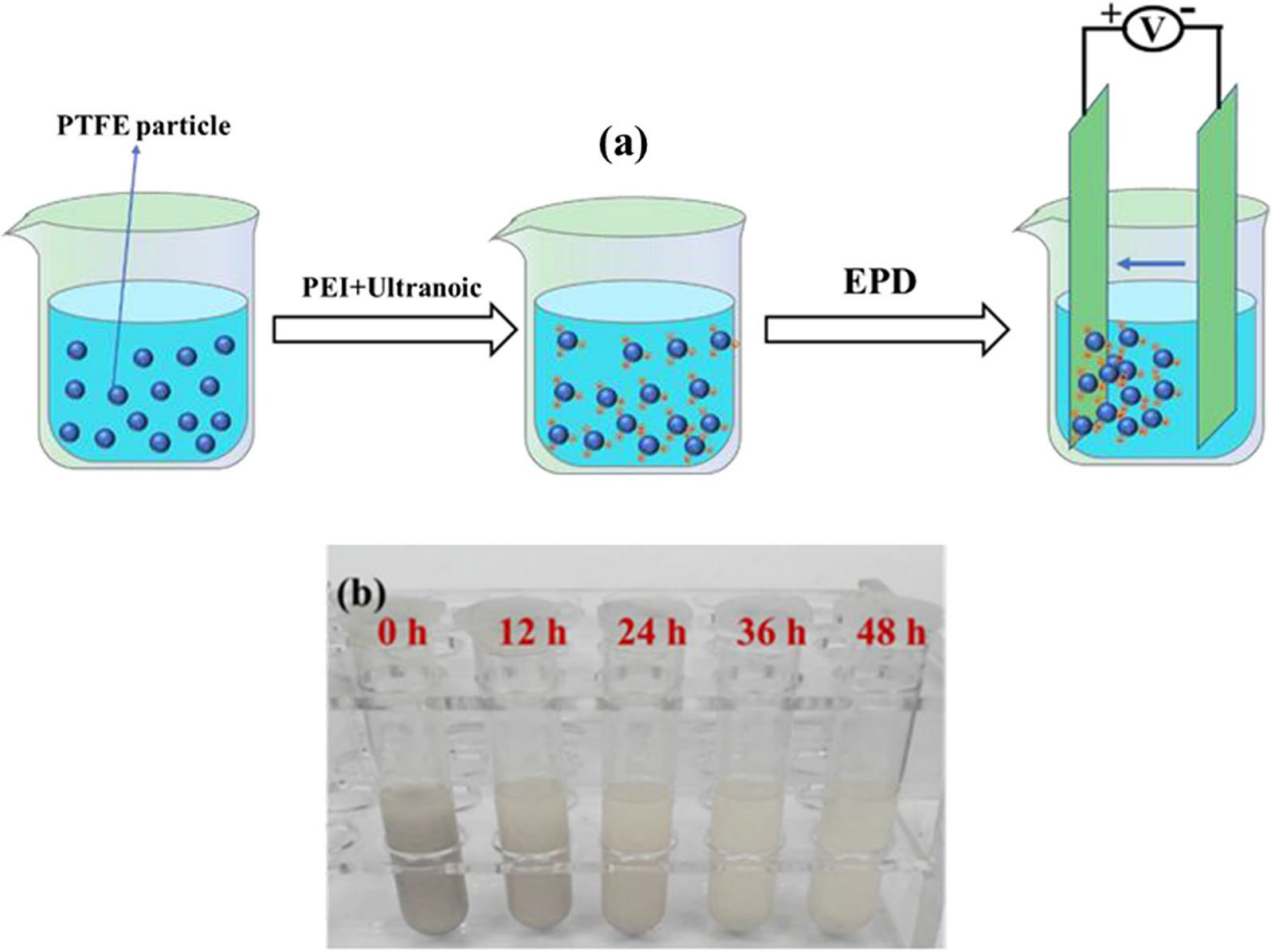
**a** Schematic diagram of PTFE coatings. **b** A series of changes in suspension stability over time

Figure 2a shows the schematic diagram of the PTFE coatings by EPD, positively charged PTFE particles in the suspension composed of ethanol, Nafion and NaOH would move to the anode in the electric field.

The suspensions were made by mixing 0.6 g PTFE particles into 100 mL ethanol solvent which contains certain 0.10 g·L^− 1^ Nafion and 0.10 mM NaOH. The suspension was then sonicated for 20 min. Next, the sonicated suspension was partially poured into the centrifuge tube and left to stand. Subsequently, at 12 h, 24 h, 36 h and 48 h respectively, conduct the same operation of preparing suspension and pouring it into the centrifuge tube for standing.

A series of changes in suspension stability over time suspension stability over time is displayed in Fig. 2b. It can be clearly seen that with the prolongation of time, the PTFE particles in the suspension settled and the suspension gradually became clear.

**Fig. 3:**
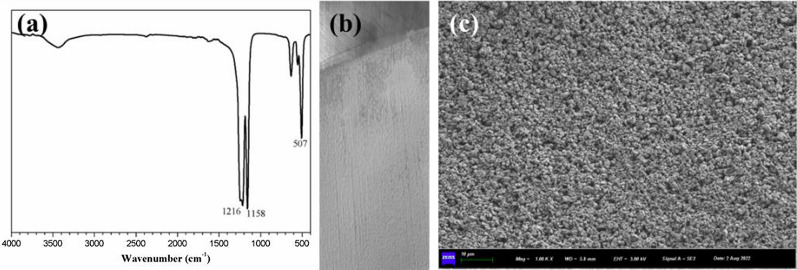
**a** FT-IR spectra of PTFE particels. **b** Image of PTFE coatings deposited AZ31 magnesium alloy sheets. **c** SEM images of PTFE coatings

The FT-IR spectra of the PTFE are shown in Fig. 3a. The spectra peaks at 1216 cm^− 1^ and 1158 cm^− 1^, which corresponded to the characteristic peaks of PTFE [21–23]. Figure 3b displays the pictures of PTFE coatings. It means the PTFE coatings have been successfully fabricated through EPD in ethanol with additive of 0.05 g·L^− 1^ Nafion and 0.05 mM NaOH. The surface of PTFE coatings is relatively fine, smooth, and uniform.

The SEM image of the morphology of the coatings is shown in Fig. 3c. The coatings are dense and uniform, and the PTFE particles show a transition trend from single particles to whole particles.

### Effect of additive concentration on electrophoretic deposition rate

It is well known that successful EPD mainly depended on a suitable dispersion media to make specific kind of particle charged and move directionally under electric-field [17–24]. Therefore, a suitable dispersion media is the key issue for successful EPD. In order to provide a suitable dispersion mixture for EPD of specific kind of particle [25], the dispersion mixture needs to be optimized case by case. In this work, we introduced NaOH and Nafion as additive scheme for EPD of PTFE. Since Nafion is expensive, the addition of additive NaOH can effectively reduce the amount of additive Nafion, thus effectively reducing the cost of EPD.

**Fig. 4:**
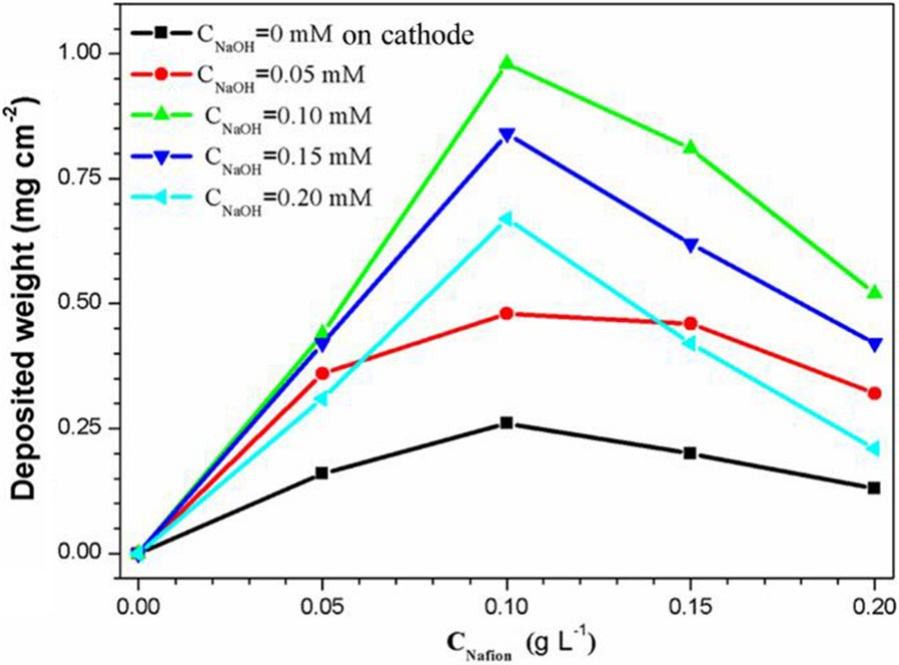
Deposition weight of the PTFE coating versus the addition amount of NaOH with different Nafion contents (0, 0.05, 0.10, 0.15 and 0.20 g·L^− 1^ Nafion)

Nafion and NaOH were both added to the PTFE ethanol suspension, and the deposition weight versus addition amount of NaOH with different Nafion amounts are shown in Fig. 4. The voltage of EPD is 60 V, and the concentration of PTFE is fixed at 6 g·L^− 1^. Four conclusions can be drawn from this observation: (1) adding Nafion alone can only realize the cathodic EPD of PTFE, and the deposition rate will increase at first and then gradually decrease as Nafion content increase; (2) adding NaOH alone cannot realize the EPD of PTFE; (3) for the Nafion-assisted EPD of PTFE, simultaneously adding NaOH can turn cathodic EPD into anodic EPD; and (4) under different content of Nafion, when the Nafion-assisted EPD of PTFE changes from cathodic into anodic as NaOH is added, the deposition rate of PTFE on the anode would increase at first and then decrease with further increasing the content of NaOH. The EPD of PTFE may be improved by Nafion for the following reasons: when Nafion is dissolved and ionized in ethanol solution, it forms a polymer cation with many positively charged groups and a bunch of dispersed anions, which are then adsorbed onto the surface of particles. Therefore, Nafion can provide the excellent stability for PTFE suspension, and its molecular chain contains a large number of sulfonyls, which could increase the number of positive charges of PTFE particles and thus improve the deposition rate of PTFE coatings. The concentrations of Nafion added are 0, 0.05, 0.10, 0.15 g·L^− 1^, respectively. When the concentration of NaOH increases from 0 to 0.10 mM, the deposition rate of PTFE particles increases obviously: within 20 min, the deposition mass increases from 0.16 mg·cm^− 2^ to 0.98 mg·cm^− 2^, indicating an increase of about six times. The results show that the added NaOH can effectively increase the amount of positive charge on the surface of PTFE particles, thus the EPD of PTFE particles is obviously improved. When the concentration of NaOH is further increased to 0.20 mM, the deposition rate of PTFE particles has not been further significantly improved, which may be because the amount of NaOH is excessive at this time, and the amount of positive charge absorbed on the surface of PTFE particles has been saturated. Therefore, the deposition rate has approached the limit and cannot be further increased. To sum up, the highest deposition rate for PTFE could be achieved with the addition of 0.10 g·L^− 1^ Nafion + 0.10 mM NaOH, the results is in consistence with the conclusion drawn from the Zeta potential test. And the PTFE particles have no tendency to agglomerate and have a slow settling rate in the suspension.

**Fig. 5:**
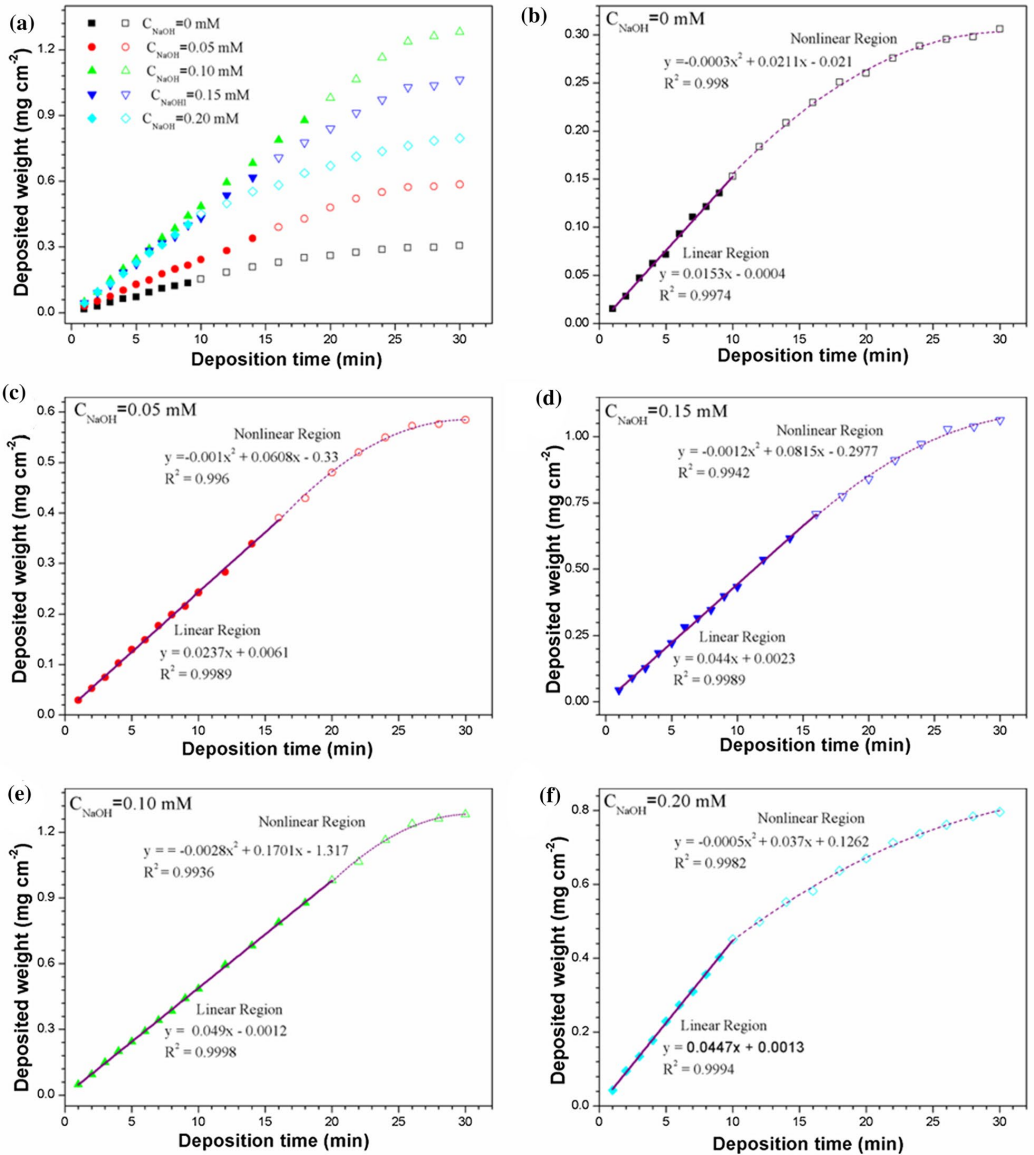
**a** Transformation law of deposited quantity over time during EPD process in different NaOH concentrations. **b**–**f** The relation between deposit quantity and deposition time for both short time period and long time period at **b** 0, **c** 0.05, **d** 0.10, **e** 0.15 and **f** 0.20 mM NaOH concentrations

Shown in Fig. 5 is the deposited weight (mg·cm^− 2^) as a function of deposition time and NaOH concentrations during EPD of PTFE deposited on AZ31 magnesium alloy sheets. The concentration of Nafion is fixed at 0.10 g·L^− 1^, the concentration of PTFE is fixed at 6 g·L^− 1^, and the voltage of EPD is 60 V.

As can be seen, with 0.10 mM’s NaOH, the deposition mass of PTFE particles is basically in linear relationship with deposition time before 20 min, and the linear relationship equation is as follows:

1 $$\text{Y}=\text{a}\text{x}$$

where Y is the deposition quality per unit area (mg·cm^− 2^), x is the deposition time (minutes), a is fitting constant.

During this period, the kinetic of EPD of PTFE particles is controlled by a linear relationship, and the EPD rate of PTFE particles basically keeps constant. When the EPD of PTFE particles begins, the number of PTFE particles in the suspension is large, which can meet the requirement of stable PTFE particle source for the deposition and growth of thin coatings on the electrode. Therefore, the quality of the prepared coatings increases steadily.

However, after EPD has lasted for more than 20 min, the deposition rate of PTFE particles decreases with time, and the relationship between deposition mass and deposition time becomes parabolic.

2 $$\text{Y}=\text{a}{\text{x}}^{2}+\text{b}\text{x}+\text{c}$$

where Y is the deposition quality per unit area (mg·cm^− 2^), x is the deposition time (minutes), a, b, and c are fitting constants. During this period, the kinetic of EPD of PTFE particles belongs to the control region of parabolic relationship. The same phenomenon has been reported by others [26–29]. This may be related to the stability of the suspension configured by PTFE particles during EPD. When the EPD progresses to a certain point, the PTFE particles in the suspension cannot provide enough PTFE particles for the growth of the coatings due to the consumption of deposition and precipitation. At this point, the deposition mass increase of the PTFE coatings begins to slow down. When the deposition time is further extended, the deposition rate and the separation rate of the PTFE particles from the matrix reach equilibrium, and the thickness of the coatings will not increase. What’s more, since the coating is soaked in the solvent for too long, it will lead to a decrease in adhesion between the coating and the matrix and a fall off may happen.

During a shorter deposition, PTFE particles is given priority to negative directional migration under the effect of electric field, although PTFE particles movement speed will lead to more collision between particles, PTFE particles have not yet been growing into large enough to aggregate and produce subsidence, so the dispersing system is relatively stable, which can provide a stable source of PTFE particles for the growth of the PTFE coatings on anode and ensure the stable deposition rate of PTFE coatings on the anode electrode. With prolonging deposition time, the colliding PTFE particles continue to grow and form large enough agglomerates for mass deposition. At this time, the stability of the dispersed system is broken [30]. Due to the consumption of sedimentation, the suspension will no longer provide PTFE stable particle source for the growth of PTFE coatings on the anode. Consequently, the deposition rate of PTFE particles on the anode electrode decreases continuously.

There is a critical transition time between the linear region and the parabolic region. This transition will last for a shorter time with other NaOH concentration, the result is in consistent with the observation in Fig. 4. Obviously, the best additive scheme has three positive effects: (1) Provide the best stability for PTFE suspension; (2) Make its deposition speed the fastest; (3) Provide better stability for the suspension in the process of EPD.

### Effect of deposition voltage on the EPD kinetics

**Fig. 6:**
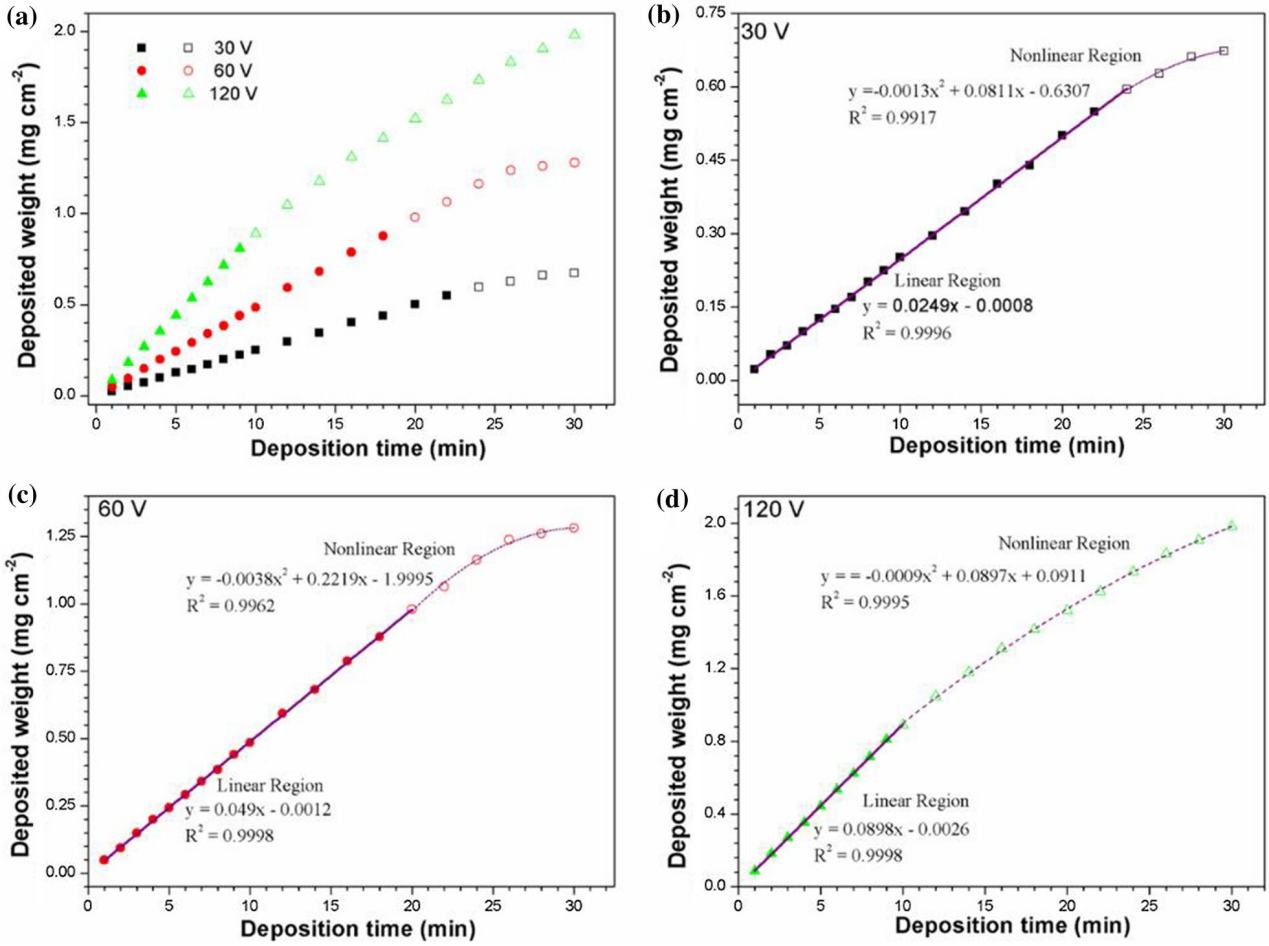
**a** Deposited quantity of PTFE coatings over time during EPD process under different field strengths. The relation between deposit quantity and deposition time for both short time period and long time period at **b** 30, **c** 60, and **d** 120 V

The EPDs of PTFE coatings on AZ31 magnesium alloy sheets are investigated under different voltage (30, 60, 120 V) as shown in Fig. 6. The concentration of Nafion is fixed at 0.10 g·L^− 1^, the concentration of NaOH is fixed at 0.10 mM, and the concentration of PTFE is fixed at 6 g·L^− 1^.

As expected, it is observed that deposition weight increased with time passing by and voltage being increased. Higher deposition voltage provides thicker coating. However, high voltage has some negative effects on the other hand: the crucial time of PTFE particles at a voltage of 120 V is shorter than that of PTFE particles at a voltage of 30 or 60 V. The reason is that under the same condition, high voltage will produce more violent hydrogen evolution reaction in the suspension [31, 32]. With the same suspension system, the current through the suspension with high voltage increases, which would lead to a rapid heating-up of the solution (the suspension can be obviously detected as hot), moreover, the non-uniformity of the collisions between PTFE particles during motion and deposition increases, therefore the suspension would become very unstable. Obviously, an unstable suspension is not beneficial to EPD process. In addition, with employing a deposition voltage of 60 V, the deposition rate was also limited by the electric drive force, which resulted in a relatively thin PTFE coating. Therefore, the deposition rate of 60 V is relatively suitable for fabricating PTFE particle coating by EPD with consideration of both deposition rate and suspension stability.

### Effect of concentration of PTFE particles on electrophoretic deposition rate

**Fig. 7:**
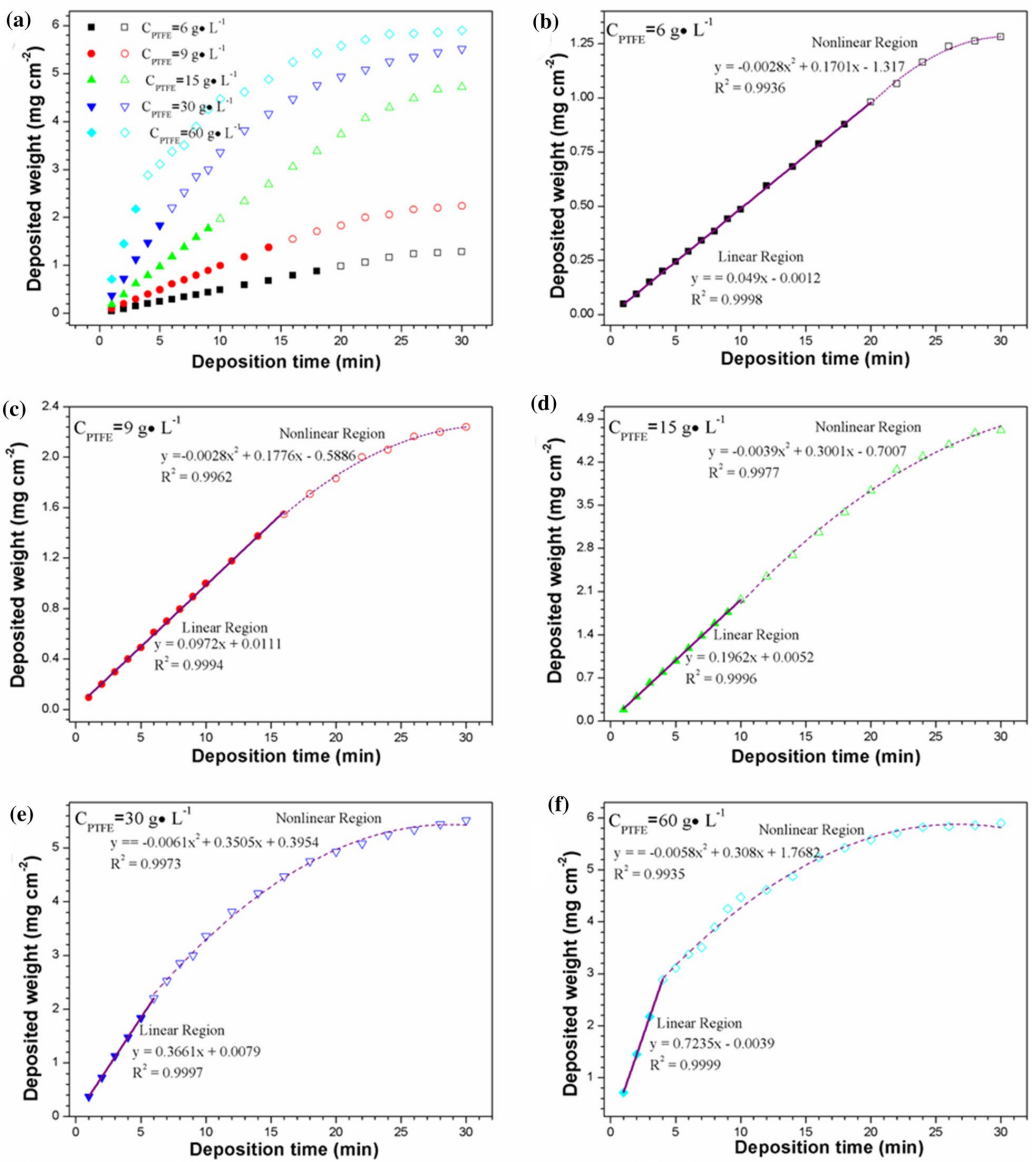
**a** Transformation law of deposited quantity over time during EPD process in different PTFE concentrations. **b**–**f** The relation between deposit quantity and deposition time for both short time period and long time period at **b** 6, **c** 9, **d** 15, **e** 30 and **f** 60 g·L^− 1^ PTFE concentrations

The EPDs of PTFE coatings on AZ31 magnesium alloy sheets are investigated under different PTFE particles concentrate (6, 9, 15, 30, 60 g·L^− 1^) as shown in Fig. 7. The concentration of Nafion is fixed at 0.10 g·L^− 1^, the concentration of NaOH is fixed at 0.10 mM. The EPD was carried out with a voltage of 60 V. The electrophoretic deposition rate of PTFE coatings increases to the maximum with the increase of the concentration of PTFE particles and then decreases. There are two reasons: (i) The quality of the PTFE particle coating is related to the mass of PTFE particles suspended in the suspension. When the concentration of PTFE particles is too high, they cannot be suspended in the suspension, but precipitate in the suspension. (ii) When the EPD of PTFE particles begins, the number of PTFE particles in the Nafion suspension is large, which can meet the requirement of stable PTFE particle source for the deposition and growth of thin PTFE coatings on the AZ31 magnesium alloy sheets electrode. Therefore, the quality of the prepared PTFE coatings increases steadily. When the electrophoretic deposition progresses to a certain point, the PTFE particles in the suspension cannot provide enough PTFE particles for the growth of the coatings due to the consumption of deposition and precipitation. At this point, the mass increase of the electrophoretic deposition coating begins to slow down. As the deposition time is prolonged, the deposition rate and the separation rate of the PTFE particles from the AZ31 magnesium alloy sheets will reach equilibrium, and the thickness of the coatings will not increase. What’s more, since the coating is soaked in the solvent for too long, it will lead to a decrease in adhesion between the coatings and the AZ31 magnesium alloy sheets and a fall off may happen.

Moreover, deposition mass decreases gradually with the prolonged deposition time. When the deposition time is more than 20 min, the relative decrease of deposition mass is the smallest when the PTFE concentration is 15 g·L^− 1^.

## Conclusion

The PTFE coatings on AZ31 magnesium alloy have been prepared by EPD method at ambient temperature and pressure. Using Nafion as a surface charging additive, PTFE particles can be deposited on the cathode substrate. Moreover, the addition of NaOH can effectively reduce the amount of additive Nafion, thus effectively reducing the cost of EPD. And the Nafion and NaOH had reached a synergistic effect in electrophoretic deposition, and the PTFE particles are deposited from cathode substrate to anode substrate. Deposition kinetics of PTFE coating have been investigated by altering voltage, regulating surface charging additive concentration and deposition time with different PTFE particle concentrations. In a word, this study provides a new method of EPD to effectively realize anodic assembling of PTFE particles on AZ31 magnesium alloy.

## Data Availability

All data generated or analyzed during this study are included in this published article.
